# Comparison of the prognostic values of ^18^F-fluorodeoxyglucose parameters from colon and non-colon sites of involvement in diffuse large B-cell lymphoma of the colon

**DOI:** 10.1038/s41598-020-69550-6

**Published:** 2020-07-29

**Authors:** Chae Hong Lim, Seung Hyup Hyun, Seung Hwan Moon, Young Seok Cho, Joon Young Choi, Kyung-Han Lee

**Affiliations:** 1 Department of Nuclear Medicine, Soonchunhyang University Hospital, Seoul, Korea; 2 Department of Nuclear Medicine, Samsung Medical Center, Sungkyunkwan University School of Medicine, 81 Irwon-ro, Gangnam-gu, Seoul, 06351 Korea

**Keywords:** Cancer imaging, Cancer metabolism

## Abstract

We examined the prognostic values of ^18^F-fluorodeoxyglucose (^18^F-FDG) parameters from colon, non-colon, and total lesions in patients with diffuse large B-cell lymphoma (DLBCL) of the colon. Positron emission tomography/computed tomography (PET/CT) in 50 patients was retrospectively analyzed for maximum standardized uptake value (SUVmax), metabolic tumor volume (MTV) and total lesion glycolysis (TLG). During follow-up, 13 patients showed progression and 9 died from disease. Receiver operating characteristics (ROC) curve analysis showed that non-colon and total lesion MTV and TLG and colon lesion SUVmax were associated with progression or death. Significant univariate predictors of poor event-free survival (EFS) included stage III-IV, greater International Prognostic Index (IPI) score, no resection, high non-colon lesion SUVmax, MTV and TLG, and high total lesion MTV and TLG. Univariate predictors of poor overall survival (OS) included stage III–IV, greater IPI score, no resection, high non-colon lesion MTV and TLG, high total lesion MTV, and low colon lesion SUVmax. Multivariate analysis revealed that high non-colon lesion TLG was independently associated with poor EFS and OS. Low colon lesion SUVmax was also independently associated with poor OS. In a subgroup with colon-dominant involvement (n = 35), non-colon lesion MTV and TLG were associated with events and non-colon lesion MTV was associated with patient death. Univariate analysis showed that high non-colon lesion MTV was a significant predictor of poor EFS and OS, while non-colon lesion TLG was a significant predictor of poor OS. Thus, volumetric FDG parameters of non-colon lesions offered significant prognostic information in patients with DLBCL of the colon.

## Introduction

Primary lymphoma of the colon is a rare tumor of the gastrointestinal tract that comprises only 0.2–1.2% of all colonic malignancies. The most common type of colonic lymphoma is diffuse large B-cell lymphoma (DLBCL)^1,2^. Primary colon DLBCL is similar to other types of DLBCL in that it is generally treated using combination chemotherapy with rituximab, cyclophosphamide, doxorubicin, vincristine and prednisone (R-CHOP)^3,4^. However, while gastrointestinal DLBCLs respond more favorably to CHOP chemotherapy compared to nodal disease^5^, they are less responsive to rituximab treatment^6,7^. Meanwhile, primary colon DLBCL shares certain traits with epithelial colon cancer. For example, selected patients exhibit a survival benefit from colon lesion resection^8,9^. In addition, colon DLBCL can be classified with the TNM system, similar to that of colon cancer^10^.

Several lines of evidence imply that extranodal non-Hodgkin lymphomas (NHL) behave divergently from their nodal counterparts. Indeed, biologic characterization indicates that extranodal NHLs should be regarded as separate nosological entities with a unique genetic origin compared to nodal disease. Consistent with this notion, gastrointestinal NHLs have a different dissemination pattern and clinical outcome compared to nodal disease^10–13^. This has led to the development of newer prognostic parameters for primary gastrointestinal DLBCL to improve outcome stratification and help select the optimal mode of management. However, this has been difficult for primary colon DLBCL, because of its relative rarity compared to gastric and small bowel DLBCL^14,15^.

^18^F-fluorodeoxyglucose positron emission tomography/computed tomography (FDG PET/CT) is widely used for the diagnosis and staging of DLBCL. Furthermore, FDG uptake of lesions quantified as maximum standard uptake value (SUVmax), metabolic tumor volume (MTV) or total lesion glycolysis (TLG) offers important prognostic information^16–19^. In patients with epithelial colon cancer, these prognostic parameters are mainly obtained from the primary tumor lesion^20^. In contrast, FDG parameters in patients with lymphoma are routinely measured from all detectable lesions. This is in part because the primary lesion is not discernable in nodal lymphomas including DLBCL^16,18–20^. However, the site of origin may be readily identified in primary extranodal DLBCL. In these patients, therefore, FDG parameters obtained from the primary and disseminated sites might offer distinct prognostic values.

In this study, we investigated patients with lower gastrointestinal symptoms who were diagnosed with colon DLBCL by colonoscopic biopsy. Pretreatment FDG PET/CT-based SUVmax, MTV and TLG of the colon lesion, non-colon lesions, and total lesions were analyzed for associations with patient survival. Analysis was also performed in subjects restricted to those with colon-dominant involvement as a more stringent criterion for primary colon DLBCL.

## Materials and methods

### Study population

We reviewed 241 consecutive patients with colonoscopic biopsy-confirmed lymphoma of the colon who underwent FDG PET/CT at our institution between January 2008 and October 2017. Pathology reports showed that 132 cases had DLBCL. After excluding 57 subjects with recurrent disease, 75 patients with newly diagnosed colon DLBCL were selected. Among these subjects, we excluded 23 cases whose PET/CT was performed post-treatment, one case with early follow-up loss, and one case with a coexisting second malignancy. Consequently, a total of 50 patients were finally included in this study.

All of the study subjects presented with serious lower gastrointestinal symptoms including abdominal pain, gastrointestinal bleeding or palpable abdominal mass that required colonoscopy. This led to diagnosis of DLBLC via colon lesion biopsy. All subjects also underwent esophagogastroduodenoscopy, pharyngeal examination, chest CT, abdomen and pelvis CT and bilateral iliac crest bone marrow biopsy. In all cases, the findings of these tests satisfied the definition of primary gastrointestinal lymphoma as proposed by Krol et al. ^21^, and the colon lesion was of primary clinical importance in all subjects. Furthermore, additional analyses were also performed in a subgroup of subjects with colon-dominant involvement as a more stringent criterion for primary colon DLBCL. These subjects either had only colon lesions or colon lesions with MTV and TLG greater than all non-colon lesions.

This retrospective observational study was approved by our institutional review board and the need for written consent from study subjects was waived.

### FDG PET/CT imaging

All patients fasted for at least 6 h and had blood glucose < 150 mg/dl at the time of PET/CT. Imaging was performed 60 min after injection of 5 MBq/kg FDG (mean ± SD, 328.9 ± 65.3 MBq; range, 218.0–488.0 MBq) without intravenous or oral contrast on a Discovery LS (GE Healthcare; n = 6) or a Discovery STe PET/CT scanner (GE Healthcare; n = 44). Continuous spiral CT was performed with an 8-slice helical CT (140 keV; 40–120 mA; Discovery LS) or 16-slice helical CT (140 keV; 30–170 mA; Discovery STe). For Discovery LS, emission scans were obtained for 4 min per frame in 2-D mode with reconstruction of attenuation-corrected PET images (4.3 × 4.3 × 3.9 mm) using an ordered-subset expectation maximization algorithm (28 subsets, 2 iterations). For Discovery STe, emission scans were 2.5 min per frame in 3-D mode with reconstruction of attenuation-corrected PET images (3.9 × 3.9 × 3.3 mm) using a 3-D ordered-subset expectation maximization algorithm (20 subsets, 2 iterations).

### Review of PET images and analysis of FDG uptake

PET images were analyzed independently of subject survival outcome by Dr. Lim CH, a nuclear medicine physician with more than 5 years of experience in interpreting oncologic FDG PET/CT studies, who also performed selection of study subjects. Metabolic parameters of lesions were measured on a GE Advantage Workstation version 4.4 using Volume Viewer Software that allows automatic volume determination by an SUV-based iso-contour threshold method. Measurements were done for the colon lesion and all non-colon lesions. The latter was any lesion outside the colon including regional nodes. Total lesions included both the colon lesion and all non-colon lesions. SUVmax was measured as the highest SUV in the colon lesion, among all non-colon lesions, and among total lesions. Non-colon lesion SUVmax was assigned a value of 1.0 if there was no detectable lesion on PET/CT. MTV was delineated with a 41% SUVmax threshold as proposed by Meignan et al.^22^. TLG was the sum of the product of MTV and SUVmean in each lesion^22^. MTV and TLG were determined for the colon lesion, for all non-colon lesions, and for total lesions.

### Medical record review

Medical records were reviewed for clinical characteristics, including age, sex, Eastern Cooperative Oncology Group (ECOG) performance status, B symptoms, serum lactate dehydrogenase (LDH) level and involvement site. The International Prognostic Index (IPI) was calculated using these data and Ann Arbor stage. Event-free survival (EFS) was defined as the time from initial diagnosis to progression, recurrence, death, or last follow-up. Overall survival (OS) was defined as the time from initial diagnosis to death or last follow-up.

### Statistical analyses

Receiver operating characteristics (ROC) curve analysis was performed to identify optimal cutoffs of FDG parameters for event prediction. Significant FDG parameters were included for survival analyses. Clinical variables included age at diagnosis, sex, Ann Arbor stage, LDH elevation, B symptom, IPI score, and surgical resection. Univariate analysis for significant prognostic factors was performed by the Kaplan–Meier method with log rank tests. Significant univariate variables (*P* < 0.05) were included for multivariate analysis using the Cox proportional hazards regression model. All statistical tests were two-sided with a significance level set at 0.05 and were performed with SPSS 23.0 (SPSS Inc., Chicago, IL, USA) or MedCalc 15.5 (MedCalc, Mariakerke, Belgium).

## Results

### Clinical and pathological features

Baseline demographic and clinical characteristics of the 50 study subjects are summarized in Table 1 and Supplementary table 1. The median age was 56 years (range, 25–85 years), and the male to female ratio was 1.17:1. B symptoms were present in 12 patients (24.0%) and serum LDH was increased in 19 patients (38.0%). Most subjects had good performance (n = 47; 94%) and localized disease with a low IPI score (n = 39, 78%). The involved colon region included the ileocecal region in 46 cases (92%; ileocecal only in 42 cases), only the ascending colon in 3 cases, and the transverse and descending colon in 1 case.

**Table 1:** Patient clinical characteristics at presentation.

Characteristic	No. of patients
Age, > 60	21 (42%)
Sex, male	27 (54%)
Performance status, ECOG >1	3 (6%)
B-symptoms at presentation	12 (24%)
Involvement site features	
Confined to colon	10 (20%)
Colon & regional LNs	16 (32%)
Colon & intra-abdominal distant LNs	10(20%)
Colon & extra-abdominal LNs	4 (8%)
Disseminated spread to extra-nodal sites†	10 (20%)
Colon involvement sites	
Ileocecal lesion*	46 (92%)
Non-ileocecal lesion	4 (8%)
Ann Arbor stage	
I–II	36 (72%)
III–IV	14 (28%)
LDH > upper normal range	19 (38%)
IPI	
Low or low-intermediate risk	39 (78%)
Intermediate-high or high risk	11 (22%)
Front-line treatment	
Resection only	1 (2%)
R-CHOP #1-4	1 (2%)
R-CHOP #5-8	11 (22%)
Resection + R-CHOP #1-4	4 (8%)
Resection + R-CHOP #5-8	33 (66%)

ECOG**,** Eastern Cooperative Group; LN, lymph node; LDH, lactate dehydrogenase; IPI, International Prognostic Index; R-CHOP, rituximab-cyclophosphamide-hydroxydaunorubicin-vincristine-prednisone; †, Extra-nodal sites included other colon, peritoneum, liver, and bone marrow.

*4 cases also included non-ileocecal lesions.

Lymphoma was confined to the colon in 10 cases (20%) and the colon and regional lymph nodes (LNs) in 16 cases (32%). Together, these 26 cases satisfied the original definition of primary colon DLBCL as proposed by Dawson et al.^23^. All of these cases underwent surgical resection with regional LN dissection, which confirmed the absence or presence of spread to pericolic LNs and colic LNs along the mesocolic borders.

Ten subjects (20%) had involvement of intra-abdominal distant LNs including retroperitoneal, upper abdominal, iliac or inguinal LNs. Four had stage III disease (8.0%) and 10 had stage IV disease (20%). Together, these 24 cases were considered to have disseminated disease, and met the broader criteria for primary colon lymphoma according to Krol et al. ^21^. Extranodal sites outside the colon were involved in 7 patients (14.0%), including 1 case with biopsy-confirmed bone marrow involvement.

### Patient outcomes

Patients were treated by colon lesion resection alone in 1, surgical resection followed by R-CHOP in 37, and R-CHOP chemotherapy alone in 12 cases. Surgical resection was performed in 34 of 36 subjects with stage I–II disease (94.4%); 2 cases refused operation. Surgical resection was also performed in 4 of 14 subjects with stage III–IV disease (28.6%) for complications such as intestinal perforation. After first-line treatment, 47 patients (94%) achieved complete remission. After a median follow-up of 39 months (range, 3–124 months), 13 patients had relapse or progression of disease and 9 patients died. One patient with stage IV disease died after 2 cycles of R-CHOP. The 5-year EFS for the entire population was 75.5% and 5-year OS was 79.3%.

### FDG parameters for outcome and optimal cut-off values

Results of ROC analyses for outcome prediction and optimal cut-off values of FDG parameters are summarized in Table 2. Colon lesion SUVmax (area under the curve [AUC] = 0.703; *P* = 0.008), non-colon lesion MTV (AUC = 0.867; *P* = 0.001) and TLG (AUC = 0.863; *P* = 0.001), and total lesion MTV (AUC = 0.782; *P* = 0.001) and TLG (AUC = 0.715; *P* = 0.007) were significant FDG parameters for predicting the occurrence of events. The optimum cutoffs for these parameters were 7.8, 56.5 cm^3^, 636.1 cm^3^, 80.0 cm^3^ and 1,288.7 cm^3^, respectively.

**Table 2:** ROC analyses of PET/CT parameters for predicting survival outcomes.

Variable	Median (range)	Event			Death		
		AUC	Cut-off	*P*	AUC	Cut-off	*P*
**Total population (n = 50)**							
Colon lesion							
SUVmax	23.4 (19.4–28.2)	0.652	14.5	0.105	0.725	15.7	0.014
MTV	47.8 (16.7–137.0)	0.568	70.0	0.457	0.512	180.0	0.905
TLG	585.4 (176.8–1919.2)	0.518	2,165.2	0.847	0.580	2,165.2	0.380
Non-colon lesion							
SUVmax	11.8 (1.0–22.0)	0.703	7.8	0.008	0.610	1.0	0.210
MTV	7.1 (0–62.3)	0.867	56.5	0.001	0.810	62.3	0.001
TLG	49.3 (0–713.4)	0.863	636.1	0.001	0.794	830.2	0.001
Total lesion							
SUVmax	23.8 (19.4–29.7)	0.602	16.2	0.314	0.663	16.2	0.145
MTV	90.5 (26.7–221.6)	0.782	80.0	0.001	0.748	80.0	0.010
TLG	1,236.5 (425.3–2,972.9)	0.715	1,288.7	0.007	0.661	1,288.7	0.091
**Colon lesion-dominant subgroup (n = 35)**							
Colon lesion							
SUVmax	24.3 (20.8–30.3)	0.637	14.5	0.347	0.786	14.5	0.108
MTV	48.6 (17.7–144.8)	0.640	70.0	0.293	0.510	70.0	0.950
TLG	974.0 (220.5–2,662.7)	0.567	176.8	0.594	0.604	1,285.2	0.368
Non-colon lesion							
SUVmax	7.4 (1.0–18.5)	0.707	1.0	0.039	0.542	1.0	0.658
MTV	1.6 (0–9.4)	0.813	1.6	0.001	0.668	1.6	0.049
TLG	7.1 (0–65.4)	0.793	7.1	0.001	0.625	7.1	0.149
Total lesion							
SUVmax	24.3 (20.8–30.3)	0.630	14.5	0.379	0.786	14.5	0.108
MTV	56.0 (19.4–166.8)	0.680	73.05	0.188	0.510	73.05	0.950
TLG	1,022.1 (228.1–2,866.3)	0.587	176.8	0.511	0.604	1,311.9	0.368

ROC, Receiver operating characteristics; AUC, Area under the curve; SUVmax, Maximum standard uptake value; MTV, Metabolic tumor volume; TLG, Total lesion glycolysis; Non-colon lesion SUVmax was assigned a value of 1.0 if there was no detectable lesion on PET/CT.

Significant FDG parameters for predicting patient death were colon lesion SUVmax (AUC = 0.725, *P* = 0.014), non-colon lesion MTV (AUC = 0.810, *P* = 0.001) and TLG (AUC = 0.794, *P* = 0.001), and total lesion MTV AUC = 0.748, *P* = 0.010). The optimal cut-off values in this situation were 15.7, 62.3 cm^3^, 830.2 cm^3^ and 80.0 cm^3^, respectively.

### Univariate and multivariate survival analyses

Results of univariate and multivariate analyses for predictors of EFS and OS are summarized in Tables 3 and 4, respectively. Significant univariate predictors of poor EFS were stage III-IV (*P* = 0.001), increased LDH (*P* = 0.002), greater IPI score (*P* = 0.016), no resection (*P* = 0.005), high non-colon lesion SUVmax (*P* = 0.046), MTV (*P* = 0.001) and TLG (*P* = 0.001), and high total lesion MTV (*P* = 0.005) and TLG (*P* = 0.007). Univariate predictors of poor OS were stage III-IV (*P* = 0.002), increased LDH (*P* = 0.009), greater IPI score (*P* = 0.008), B symptoms (*P* = 0.026), no resection (*P* = 0.005), high non-colon lesion MTV (*P* = 0.001) and TLG (*P* = 0.001), high total lesion MTV (*P* = 0.024), and low colon lesion SUVmax (*P* = 0.010).

**Table 3:** Univariate and multivariate analysis for event-free survival (EFS).

Clinical variables	Event rate	EFS		
		HR	95% CI	*P*
**Univariate analysis**				
Age (> 60)	6/21	1.38	0.46–4.14	0.566
Male gender	7/27	1.02	0.34–3.06	0.968
Ann Arbor Stage, III-IV	9/14	7.62	2.33–24.92	0.001
LDH elevation	9/19	11.67	2.44–55.73	0.002
B symptom	6/12	2.96	0.99–8.85	0.053
IPI score 3–5	6/11	3.89	1.28–11.76	0.016
No surgical resection	8/13	5.05	1.63–15.68	0.005
Non-colon lesion SUVmax > 7.8	12/31	7.98	1.04–61.56	0.046
Non-colon lesion MTV > 56.5	10/15	10.51	2.87–38.51	0.001
Non-colon lesion TLG > 636.1	10/13	15.39	4.17–56.84	0.001
Total lesion MTV > 80.0	12/25	18.79	2.39–147.9	0.005
Total lesion TLG > 1,288.7	10/23	8.19	1.78–37.65	0.007
**Multivariate analysis**				
Non-colon lesion TLG > 636.1		15.39	4.17–56.84	0.001

HR = hazard ratio; CI = confidence interval; LDH, lactate dehydrogenase; IPI, International Prognostic Index; SUVmax, Maximum standard uptake value; MTV, Metabolic tumor volume; TLG, Total lesion glycolysis.

**Table 4:** Univariate and multivariate analysis for overall survival (OS).

Clinical variables	Event rate	OS		
		HR	95% CI	*P*
**Univariate analysis**				
Age (> 60)	4/21	1.47	0.39–5.51	0.569
Male gender	4/27	0.74	0.20–2.78	0.658
Ann Arbor stage III–IV	7/14	12.84	2.65–62.30	0.002
LDH elevation	6/19	9.09	1.76–47.06	0.009
B symptom present	5/12	4.49	1.20–16.75	0.026
IPI score 3–5	5/11	5.94	1.58–22.28	0.008
No surgical resection	6/13	7.33	1.81–29.67	0.005
Colon lesion SUVmax ≤ 15.7	5/11	5.76	1.53–21.61	0.010
Non-colon lesion MTV > 62.3	6/12	9.94	2.44–40.52	0.001
Non-colon lesion TLG > 830.2	6/11	19.23	3.78–97.82	0.001
Total lesion MTV > 80.0	8/25	11.07	1.38–88.87	0.024
**Multivariate analysis**				
IPI score 3–5 vs. 0–2		6.76	1.12–41.05	0.038
Colon lesion SUVmax ≤ 15.7		11.53	1.95–68.03	0.007
Non-colon lesion TLG > 636.1		11.22	1.87–67.40	0.008

HR, hazard ratio; CI, confidence interval; LDH, lactate dehydrogenase; IPI, International Prognostic Index; SUVmax, Maximum standard uptake value; MTV, Metabolic tumor volume; TLG, Total lesion glycolysis.

Forward stepwise multivariate Cox proportional hazards models revealed that high non-colon lesion TLG (HR, 15.39; 95% CI, 4.17–56.84; *P* = 0.001) was the single significant independent predictor of poor EFS. Significant independent predictors of poor OS were greater IPI score (HR = 6.76; 95% CI, 1.12–41.05; *P* = 0.038), high non-colon lesion TLG (HR = 11.22; 95% CI, 1.87–67.40; *P* = 0.008), and low colon lesion SUVmax (HR = 11.53; 95% CI, 1.95–68.03, *P* = 0.007).

### Prognostic value of FDG parameters after stratification according to lymphoma stage

**Figure 1:**
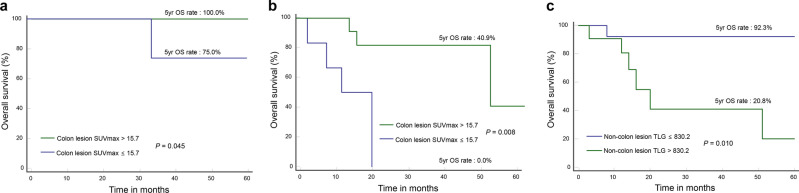
Kaplan–Meier curves for survival in patients stratified for lymphoma stage. (**a**) Overall survival in 26 patients with loco-regional disease according to colon lesion SUVmax. (**b**, **c**) Overall survival in 24 patients with disseminated disease according to colon lesion SUVmax (**b**) and non-colon lesion TLG (**c**).

Among 26 loco-regionally confined DLBCL, all had low non-colon TLG (≤ 830.2) and 22 had high colon lesion SUVmax (> 15.7). All 22 with high colon lesion SUVmax survived during follow-up, whereas 1 of 4 with low colon lesion SUVmax died. Low and high colon lesion SUVmax was associated with 75.0% and 100.0% 5-year OS, respectively. Among 24 subjects with disseminated DLBCL, both low colon lesion SUVmax (0.0% vs. 40.9%; *P* = 0.008; Fig. 1b) and high non-colon TLG (20.8% vs. 92.3%; *P* = 0.010; Fig. 1c) appeared to be associated with worse 5-year OS. However, the significance of these findings is uncertain due to small sample size.

### Prognostic value of FDG parameters after stratification according to surgery

The 12 patients who did not undergo surgical resection had a significantly worse 5-year OS compared to the 38 patients who did (41.3% vs. 93.2%; *P* = 0.001). In order to exclude the influence of surgery, we performed additional analysis after patients were stratified into resection and non-resection groups.

**Figure 2:**
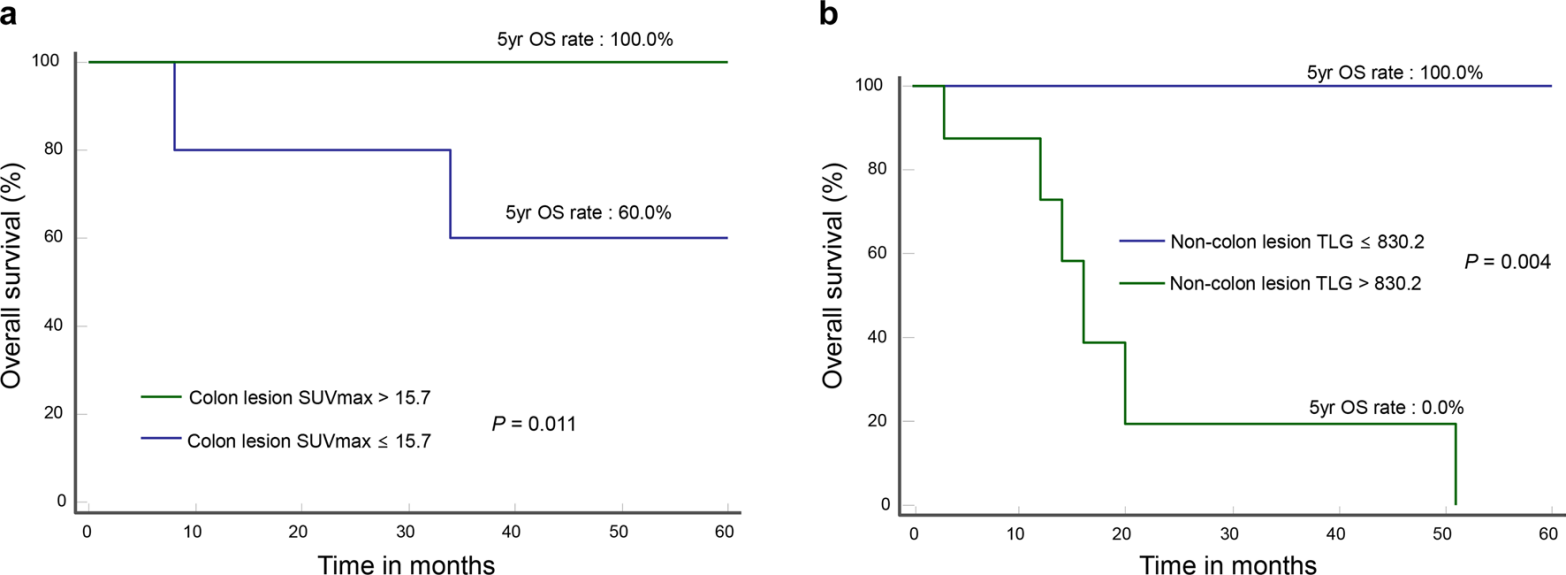
Kaplan–Meier curves for survival in patients stratified for surgical resection. (**a**) Overall survival according to colon lesion SUVmax in 38 patients with surgical resection, and (**b**) according to non-colon lesion TLG in 12 patients without surgical resection.

In the “resection group”, low colon lesion SUVmax was associated with a significantly worse 5-year OS (60.0% vs. 100.0%; *P* = 0.011; Fig. 2a), whereas high non-colon lesion TLG was not (100.0% vs. 92.8%; *P* = 0.717; data not shown). In the “non-resection group”, high non-colon lesion TLG was linked to a significantly worse 5-year OS (0.0% vs. 100.0%; *P* = 0.004; Fig. 2b), whereas lower colon lesion SUVmax was not (33.3% vs. 44.4%; *P* = 0.537).

### Prognostic value of FDG parameters after stratification according to IPI score

**Figure 3:**
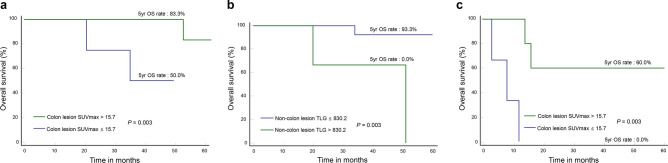
Kaplan–Meier curves for survival in patients stratified for IPI score. (**a**, **b**) Overall survival in 39 patients with low IPI score according to colon lesion SUVmax (**a**) and non-colon lesion TLG (**b**). (**c**) Overall survival in 11 patients with high IPI score according to colon lesion SUVmax.

IPI score was the single clinical variable with independent prognostic value in our study. We therefore performed additional analysis after patients were stratified according to this variable. In patients with low IPI score (0–2; n = 39), both low colon lesion SUVmax (50.0% vs. 83.3%; *P* = 0.003; Fig. 3a) and high non-colon TLG (0.0% vs. 93.3%; *P* = 0.003; Fig. 3b) appeared to be associated with worse 5-year OS. In patients with high IPI score (3–5; n = 11), low colon lesion SUVmax appeared to be linked to a worse 5-year OS (0.0% vs. 60.0%; *P* = 0.003; Fig. 3c), whereas high non-colon lesion TLG was not (20.8% vs. 80.0%; *P* = 0.112; data not shown). However, it was difficult to draw firm conclusions on these finding due to small sample size.

### Prognostic value of FDG parameters in the colon lesion-dominant subgroup

The 35 subjects with colon lesion-dominant involvement had higher colon lesion SUVmax (median value, 24.3 vs. 7.4), MTV (median value, 48.6 vs. 1.6 cm^3^) and TLG (median value, 585.4 vs. 49.3) compared to non-colon lesions (Table 2). ROC analysis in this subgroup demonstrated that non-colon lesion MTV (AUC = 0.813; *P* = 0.001) and TLG (AUC = 0.793; *P* = 0.001) were associated with events. The optimum cutoff for these parameters were 1.6 cm^3^ and 7.1, respectively. Non-colon lesion MTV with an optimal cut-off of 1.6 cm^3^ was also associated with patient death (AUC = 0.688, *P* = 0.049).

There were 5 events (14.3%) including 3 deaths (8.6%) in this subgroup. Significant univariate predictors of poor EFS were older age (*P* = 0.044), stage III-IV (*P* = 0.030), increased LDH (*P* = 0.034), high non-colon lesion MTV (*P* = 0.008) and TLG (*P* = 0.023; Supplementary Table 2). High non-colon lesion MTV (*P* = 0.038) was the single significant univariate predictor of poor OS (Supplementary Table 2).

**Figure 4:**
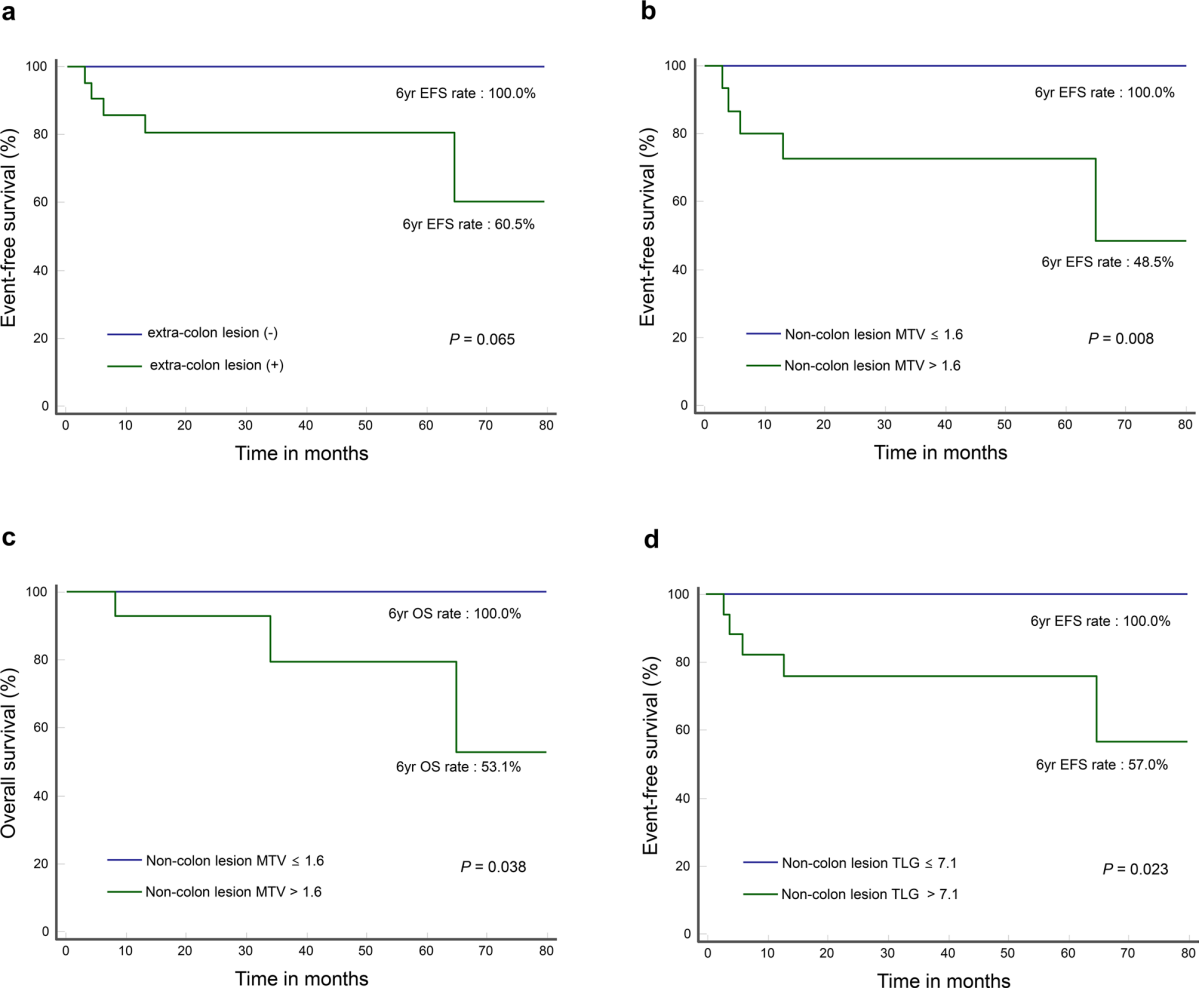
Kaplan–Meier curves for survival in a subgroup of 35 patients with colon-lesion dominant involvement. (**a**) Event free survival according to presence or absence of non-colon lesions. (**b**, **c**) Event free (**b**) and overall survival (**c**) according to non-colon lesion MTV level. (**d**) Event free survival according to non-colon lesion TLG level.

Twenty-one patients of this subgroup had non-colon lesions, which was associated with a borderline decrease in EFS (100.0% vs. 60.5%, *P* = 0.065; Fig, 4a). The non-colon lesions had high MTV in 15 cases (> 1.6), and this was associated with significantly worse EFS (48.5% vs. 100.0%; *P* = 0.008; Fig. 4b) and OS (53.1% vs. 100.0%; *P* = 0.038; Fig. 4c). The non-colon lesions had high TLG in 17 cases (> 7.1), which was linked to a significantly worse EFS (57.0% vs. 100.0%; *P* = 0.023; Fig. 4d).

## Discussion

FDG parameters from PET/CT provide valuable prognostic information in patients with various malignancies including lymphoma. However, due to its relative rarity, there are limited studies on their prognostic value in primary colon DLBCL. Furthermore, to our knowledge, this is the first study to compare the prognostic values of FDG parameters measured from the primary, metastatic, and total lymphoma lesions. In this retrospective cohort of colon DLBCL patients, we found that high non-colon lesion MTV and TLG was associated with significantly worse outcome. Although we observed an unexpected association between high colon lesion SUVmax and better patient outcome, this relation was lost when analysis was restricted to subjects with colon-dominant involvement.

Quantitative metabolic parameters derived from FDG PET/CT are gaining interest as reliable prognostic markers in various cancers^24–26^. In addition to the usefulness of primary tumor SUVmax, recent studies indicate that total body lesion MTV and TLG can provide superior prognostic information^27,28^. However, the reported relationship between quantitative FDG parameters and prognosis in patients with DLBCL has been variable^29,30^. This discordance may be contributed by different origins of DLBCL. Our study clarifies the relationship between SUVmax, MTV and TLG on EFS and OS in subjects with colon DLBCL.

In lymphomas including DLBCL, MTV and TLG are routinely derived by measuring all lesions of the body visualized by PET^17–19,22^. This is because the primary lesion cannot be discerned in nodal lymphomas. However, metastatic lesions contribute to advanced stage, and FDG parameters of disseminated sites has been reported to be able to show more prognostic values than those of primary sites^31^. Therefore, for colon lymphomas, FDG parameters from the primary and metastatic lesions might provide different prognostic information. In our study, volumetric FDG parameters of non-colon sites were independent prognostic predictors, whereas volumetric FDG parameters of the colon lesion were not. Failure of total lesion volumetric parameters to independently predict outcome is likely because this included volumetric parameters of colon lesions that had lower prognostic value.

High colon lesion SUVmax demonstrated an unexpected inverse relation with poor prognosis, which is contrary to the general notion of a positive relation in DLBCL^32,33^, as well as in colon carcinomas^20^. Gallicchio and coworkers also observed that greater SUVmax of DLBCL lesions was associated with better PFS after adjusting for other variables including MTV and TLG^34^. They suggested that this might reflect that DLBCLs with a high glycolytic rate tend to respond more readily to chemotherapy^35^. In epithelial colon carcinomas, the association between high SUVmax and worse outcome was partly contributed to cancer-associated fibroblast density^36^. It is interesting that cancer-associated fibroblasts in DLBCL were recently shown to suppress malignant cells from spreading out of lymphoid tissue^37^.

Since subjects with high colon lesion SUVmax often had locally confined disease and survived during follow-up, the link between FDG parameters and outcome was further explored after patients were stratified according to disease extent. The results confirmed that low colon lesion SUVmax remained a significant predictor of worse OS in subgroups with confined as well as disseminated DLBCL. High non-colon lesion TLG remained a significant predictor of worse OS in subjects with disseminated disease.

A potential confounding factor in our analysis is the influence of IPI score, which plays a key role on the outcome of patients with NHL^12^. This issue was explored by multivariate analysis, which demonstrated that high non-colon lesion TLG and low colon lesion SUVmax were independent predictors of poor OS after adjusting for other variables including IPI score. Furthermore, repeated analysis after stratification for IPI score showed that colon lesion SUVmax and non-colon lesion TLG were prognostic factors in both low and high IPI score groups.

Another potential confounding factor could be the performance of surgical resection, which significantly improved survival in our subjects, consistent with findings in previous reports ^8,9^. However, multivariate analysis and patient stratification again demonstrated that colon lesion SUVmax and non-colon lesion TLG were prognostic factors independent of surgical resection.

Dawson et al. proposed an early diagnostic criterion for primary colon lymphoma that required local confinement to the colon and regional nodes^24^. However, this is probably an excessively strict criterion, given that primary extranodal NHLs often spread beyond regional LNs. More recently, Krol et al. proposed that all NHLs with a dominant extranodal lesion regardless of the absence or presence of disseminated lesions should be considered primary extranodal^21^. Our study subjects are in accordance with this latter criterion. Furthermore, the colon lesions were of primary clinical importance in all subjects, who presented with severe lower gastrointestinal symptoms that led to colonoscopy and colon lesion biopsy. In addition, the clinical characteristics of our subjects are consistent with the recognized features of patients with primary colon DLBCL, including predominant male sex, fifth decade of life, and lesions in the ileocecal or ascending colon^38,39^. However, it can still be pointed out that some of our subjects might actually have nodal DLBCL with secondary colonic involvement. We therefore performed additional analysis in a subgroup population with colon-dominant involvement defined as either having colon lesion only or colon lesions with MTV and TLG greater that those of all non-colon lesions.

Analysis restricted to subjects with colon-dominant involvement confirmed the significant prognostic values of volumetric FDG parameters of non-colon sites. However, there was no association between colon lesion SUVmax and survival in this group, indicating that at least some of the remaining subjects (who had non-colon lesion TLG greater than colon lesion TLG) might have actually had nodal DLBCL with secondary colonic involvement. In the presence of such cases, the known better prognosis of primary colon DLBCL with minimal nodal involvement compared to nodal DLBCL with secondary colonic involvement would have contributed to our observed association between low colon lesion SUVmax and worse prognosis in the total study population^21^. Together, our results demonstrate that in patients with colonic DLBCL, FDG uptake of non-colon lesions make greater contribution to patient outcome than FDG uptake of the colon lesion.

This study has limitations. A major limitation its retrospective design and the small number of subjects included because of the rarity of this tumor. There is also the possibility that some of our study cases had secondary colon involvement from nodal DLBCL rather than primary colon involvement. This potential issue was addressed by subgroup analysis of subjects with colon-dominant involvement. Another limitation is the presence of different lymphoma stages and treatment modes. Although stratification analyses were performed to exclude the potential influence of these factors, lack of homogeneity might have influenced the results as confounding factors. It should also be mentioned that tumor SUV can be influenced by various factors including image resolution, reconstruction method, noise, interval between tracer injection and imaging, attenuation correction, normalization factors and plasma glucose level^40^. In our study, the use of two different PET/CT scanners is therefore a potential source for variability in SUV measurement.

## Conclusion

In patients with colon DLBCL presenting with lower gastrointestinal symptoms that required colonoscopy and biopsy, volumetric FDG parameters of non-colon lesions offered significant independent prognostic information. This was also true when analysis was restricted to a subgroup of subjects with colon-dominant involvement as a more stringent criterion for primary colon DLBCL. Thus, further studies in a larger number of subjects are warranted to confirm the prognostic value of this noninvasive imaging biomarker in DLBCL of the colon.

## Supplementary information

Supplementary information

## Data Availability

All data generated or analyzed during this study are included in this published article.

## References

[1] Wong MT, Eu KW (2006). Primary colorectal lymphomas. Colorectal Dis. Off. J. Assoc. Coloproctol. Great Britain and Ireland.

[2] Bairey O, Ruchlemer R, Shpilberg O (2006). Non-Hodgkin's lymphomas of the colon. Israel Med. Assoc. J. IMAJ.

[3] Feugier P (2005). Long-term results of the R-CHOP study in the treatment of elderly patients with diffuse large B-cell lymphoma: a study by the Groupe d'Etude des Lymphomes de l'Adulte. J. Clin. Oncol. Off. J. Am. Soc. Clin. Oncol..

[4] Habermann TM (2006). Rituximab-CHOP versus CHOP alone or with maintenance rituximab in older patients with diffuse large B-cell lymphoma. J. Clin. Oncol. Off. J. Am. Soc. Clin. Oncol..

[5] Lopez-Guillermo A (2005). Diffuse large B-cell lymphoma: clinical and biological characterization and outcome according to the nodal or extranodal primary origin. J. Clin. Oncol. Off. J. Am. Soc. Clin. Oncol..

[6] Gutierrez-Garcia G (2010). Clinico-biological characterization and outcome of primary nodal and extranodal diffuse large B-cell lymphoma in the rituximab era. Leukemia Lymphoma.

[7] Jang G (2011). Addition of rituximab to the CHOP regimen has no benefit in patients with primary extranodal diffuse large B-cell lymphoma. Korean J. Hematol..

[8] Lee J (2007). Prospective clinical study of surgical resection followed by CHOP in localized intestinal diffuse large B cell lymphoma. Leuk. Res..

[9] Kim SJ (2011). Comparison of treatment strategies for patients with intestinal diffuse large B-cell lymphoma: surgical resection followed by chemotherapy versus chemotherapy alone. Blood.

[10] Ruskone-Fourmestraux A, Dragosics B, Morgner A, Wotherspoon A, De Jong D (2003). Paris staging system for primary gastrointestinal lymphomas. Gut.

[11] Musshoff K, Schmidt-Vollmer H (1975). Proceedings: prognosis of non-Hodgkin's lymphomas with special emphasis on the staging classification. Zeitschrift fur Krebsforschung und klinische Onkologie Cancer Res Clin. Oncol..

[12] A predictive model for aggressive non-Hodgkin's lymphoma. *The New England journal of medicine* **329**, 987–994 (1993). 10.1056/NEJM199309303291402 8141877

[13] Rohatiner A (1994). Report on a workshop convened to discuss the pathological and staging classifications of gastrointestinal tract lymphoma. Ann. Oncol. Off. J. Eur. Soc. Med. Oncol..

[14] Bautista-Quach MA, Ake CD, Chen M, Wang J (2012). Gastrointestinal lymphomas: morphology, immunophenotype and molecular features. J. Gastrointest. Oncol..

[15] Ghimire P, Wu GY, Zhu L (2011). Primary gastrointestinal lymphoma. World J. Gastroenterol..

[16] Sasanelli M (2014). Pretherapy metabolic tumour volume is an independent predictor of outcome in patients with diffuse large B-cell lymphoma. Eur. J. Nucl. Med. Mol. Imaging.

[17] Esfahani SA (2013). Baseline total lesion glycolysis measured with (18)F-FDG PET/CT as a predictor of progression-free survival in diffuse large B-cell lymphoma: a pilot study. Am. J. Nucl. Med. Mol. Imaging.

[18] Song MK (2012). Clinical significance of metabolic tumor volume by PET/CT in stages II and III of diffuse large B cell lymphoma without extranodal site involvement. Ann. Hematol..

[19] Kim TM (2013). Total lesion glycolysis in positron emission tomography is a better predictor of outcome than the International Prognostic Index for patients with diffuse large B cell lymphoma. Cancer.

[20] Shi D (2015). The preoperative SUVmax for (18)F-FDG uptake predicts survival in patients with colorectal cancer. BMC Cancer.

[21] Krol AD (2003). Primary extranodal non-Hodgkin's lymphoma (NHL): the impact of alternative definitions tested in the Comprehensive Cancer Centre West population-based NHL registry. Ann. Oncol. Off. J. Eur. Soc. Med. Oncol..

[22] Meignan M (2014). Metabolic tumour volumes measured at staging in lymphoma: methodological evaluation on phantom experiments and patients. Eur. J. Nucl. Med. Mol. Imaging.

[23] Dawson IM, Cornes JS, Morson BC (1961). Primary malignant lymphoid tumours of the intestinal tract. Report of 37 cases with a study of factors influencing prognosis. Br. J. Surg..

[24] Im HJ (2015). Prognostic value of volumetric parameters of (18)F-FDG PET in non-small-cell lung cancer: a meta-analysis. Eur. J. Nucl. Med. Mol. Imaging.

[25] Lee JW (2017). Volumetric parameters on FDG PET can predict early intrahepatic recurrence-free survival in patients with hepatocellular carcinoma after curative surgical resection. Eur. J. Nucl. Med. Mol. Imaging.

[26] Paidpally V (2014). FDG volumetric parameters and survival outcomes after definitive chemoradiotherapy in patients with recurrent head and neck squamous cell carcinoma. AJR Am. J. Roentgenol..

[27] Zhang H, Wroblewski K, Appelbaum D, Pu Y (2013). Independent prognostic value of whole-body metabolic tumor burden from FDG-PET in non-small cell lung cancer. Int. J. Comput. Assist. Radiol. Surg..

[28] Zhang C (2015). Relationship between overall survival of patients with non-small cell lung cancer and whole-body metabolic tumor burden seen on postsurgical fluorodeoxyglucose PET images. Radiology.

[29] Adams HJ (2015). Prognostic superiority of the national comprehensive cancer network international prognostic index over pretreatment whole-body volumetric-metabolic FDG-PET/CT metrics in diffuse large B-cell lymphoma. Eur. J. Haematol..

[30] Alagoz E (2017). Uptake patterns of untreated primary gastrointestinal extranodal lymphomas on initial staging (18)F-FDG PET/CT and metabolic tumor parameters. Mol. Imaging Radionucl. Ther..

[31] Jin F (2018). Prognostic value of metabolic parameters of metastatic lymph nodes on (18)F-FDG PET/CT in patients with limited-stage small-cell lung cancer with lymph node involvement. Clin. Lung Cancer.

[32] Oh MY (2012). Clinical significance of standardized uptake value and maximum tumor diameter in patients with primary extranodal diffuse large B cell lymphoma. Korean J. Hematol..

[33] Chihara D (2011). High maximum standard uptake value (SUVmax) on PET scan is associated with shorter survival in patients with diffuse large B cell lymphoma. Int. J. Hematol..

[34] Gallicchio R (2014). F-18 FDG PET/CT quantization parameters as predictors of outcome in patients with diffuse large B-cell lymphoma. Eur. J. Haematol..

[35] Spaepen K (2001). Prognostic value of positron emission tomography (PET) with fluorine-18 fluorodeoxyglucose ([18F]FDG) after first-line chemotherapy in non-Hodgkin's lymphoma: is [18F]FDG-PET a valid alternative to conventional diagnostic methods?. J. Clin. Oncol. Official J. Am. Soc. Clin. Oncol..

[36] Shangguan C (2018). Cancer-associated fibroblasts enhance tumor (18)F-FDG uptake and contribute to the intratumor heterogeneity of PET-CT. Theranostics.

[37] Haro M, Orsulic S (2018). A Paradoxical correlation of cancer-associated fibroblasts with survival outcomes in B-cell lymphomas and carcinomas. Front. Cell Dev. Biol..

[38] Fan CW (2000). Primary colorectal lymphoma. Dis. Colon Rectum.

[39] Zighelboim J, Larson MV (1994). Primary colonic lymphoma. Clinical presentation, histopathologic features, and outcome with combination chemotherapy. J. Clin. Gastroenterol..

[40] Westerterp M (2007). Quantification of FDG PET studies using standardised uptake values in multi-centre trials: effects of image reconstruction, resolution and ROI definition parameters. Eur. J. Nucl. Med. Mol. Imaging.

