# Supplementary material for: Comparison of the prognostic values of 18F-fluorodeoxyglucose parameters from colon and non-colon sites of involvement in diffuse large B-cell lymphoma of the colon
Source: Sci Rep. 2020 Jul 29;10:12748. doi: 10.1038/s41598-020-69550-6 (PMC7391696; doi:10.1038/s41598-020-69550-6)
Supplement: Supplementary file 1 — Supplementary information [file 41598_2020_69550_MOESM1_ESM.pdf]

Comparison of the Prognostic Values of ^{18}F-fluorodeoxyglucose Parameters from Colon and Non-colon Sites of Involvement in Diffuse Large B-Cell Lymphoma of the Colon

Chae Hong Lim, MD[1], Seung Hyup Hyun, MD[2], Seung Hwan Moon, MD[2],
Young Seok Cho, MD[2], Joon Young Choi, MD[2], Kyung-Han Lee, MD[2]

[1]Department of Nuclear Medicine, Soonchunhyang University Hospital, Seoul, Korea
[2]Department of Nuclear Medicine, Samsung Medical Center, Sungkyunkwan University School of Medicine, Korea

SUPPLEMENTARY TABLE 1

Specific involvement sites and subgroup classification by two criteria.

Criteria	Stage	Specific involved sites	Number
Colon dominant involvement (Stringent criterion)	I	Confined colon	10
	I/II	Colon + regional LN	16
Disseminated Disease	II	Colon + abdomen LN	10
	III	Colon + LNs above/below the diaphragm	4
	IV	Multiple colon + abdomen LN	2
		Multiple colon + abdomen LN + Peritoneum	2
		Multiple colon + LNs above/below the diaphragm	1
		Colon + liver + LNs above/below the diaphragm	2
		Colon + abdomen LN + Peritoneum	1
		Colon + peritoneum + LNs above/below the diaphragm	1
		Colon + BM + LNs above/below the diaphragm	1
			50

SUPPLEMENTARY TABLE 2

Univariate analysis for event-free survival (EFS) and overall survival (OS) in colon lesion-dominant subgroup

Clinical variables	EFS				OS			
	Event rate	HR	95% CI	*P*	Event rate	HR	95% CI	*P*
Age (>60)	4/16	6.66	1.09-40.70	0.044	2/16	4.71	0.39-55.98	0.150
Male gender	2/19	0.65	0.11-3.76	0.631	0/19			0.115
Ann Arbor Stage, III-IV	2/4	5.69	0.33-99.33	0.030	1/4	4.46	0.10-191.10	0.175
LDH elevation	3/11	5.28	0.64-43.27	0.034	1/11	2.59	0.12-56.18	0.392
B symptom	2/6	4.16	0.33-52.71	0.053	1/6	4.67	0.10-215.27	0.154
IPI score 3-5	2/5	4.50	0.33-61.48	0.070	1/5	3.45	0.11-103.85	0.279
No surgical resection	1/3	2.27	0.12-42.60	0.448	0/3	-	-	0.577
Non-colon lesion MTV >1.6	5/15	-	-	0.008	3/15	-	-	0.038
Non-colon lesion TLG > 7.1	5/17	-	-	0.023	-	-	-	-

HR = hazard ratio; CI = confidence interval; LDH, lactate dehydrogenase; IPI, International Prognostic Index; SUVmax, Maximum standard uptake value; MTV, Metabolic tumor volume; TLG, Total lesion glycolysis
